# Reply to the letter from Damman et al.: Heart transplantation in the Netherlands: a national achievement

**DOI:** 10.1007/s12471-018-1092-6

**Published:** 2018-03-08

**Authors:** Y. Pinto

**Affiliations:** Department of Cardiology, Academic Medical Center, University of Amsterdam, Amsterdam, The Netherlands

We appreciate the response provided by colleagues Damman, Brügemann, De Boer, Erasmus and Van den Broek to the overview of heart transplantation at the UMCU [1] and accompanying editorial [2]. It is well taken that indeed the transplant programme at the UMCG in Groningen has developed into a strong and essential part of the total heart transplant programme in the Netherlands, and should be recognised as such. In that respect I would like to highlight that the authors nicely show that we are in the fortunate position that heart transplantation is indeed a national endeavour with equally important contributions from the three centres in Rotterdam, Utrecht and Groningen.
